# Application of Neurochemical Markers for Assessing Health Effects after Developmental Methylmercury and PCB Coexposure

**DOI:** 10.1155/2012/216032

**Published:** 2012-02-02

**Authors:** E. Roda, L. Manzo, T. Coccini

**Affiliations:** ^1^Laboratory of Clinical Toxicology, Salvatore Maugeri Foundation IRCCS, Institute of Pavia, 27100 Pavia, Italy; ^2^Department of Internal Medicine and Therapeutics, Division of Toxicology, University of Pavia, 27100 Pavia, Italy

## Abstract

Cholinergic muscarinic receptors (MRs) and monoamine oxidase activity (MAO-B), expressed both in brain and blood cells, were investigated in animals and exposed subjects to assess (i) MeHg (0.5–1 mg/kg/day GD7-PD7) and/or PCB153 (20 mg/kg/day GD10–GD16) effects on cerebellar MAO-B and MRs, and lymphocyte MRs, in dams and offspring 21 days postpartum; (ii) MAO-B in platelets and MRs in lymphocytes of a Faroese 7-year-old children cohort, prenatally exposed to MeHg/PCBs. *Animal Data*. MAO-B was altered in male cerebellum by MeHg, PCB153, and their combination (35%, 45%, and 25% decrease, resp.). Cerebellar MRs were enhanced by MeHg alone in dams (87%) and male pups (27%). PCB153 alone and in mixture did not modify cerebellar MRs. Similarly to brain, lymphocyte MRs were enhanced in both dams and offspring by MeHg alone. All changes were caused by 1 MeHg mg/kg/day, the lower dose was ineffective. *Human Data*. Both biomarkers showed homogeneous distributions within the cohort (MRs, range 0.1–36.78 fmol/million cells; MAO-B, 0.95–14.95 nmol/mg protein/h). No correlation was found between the two biomarkers and neurotoxicant concentrations in blood (pre- and postnatally).

## 1. Introduction

In the last few decades, the continuous exposure of humans to complex mixture of contaminating substances in food is an issue that has been giving increasing cause for concern. Clinical and experimental research has demonstrated the high vulnerability of the developing nervous system to toxic insults, leading to permanent alteration of brain functions and pathologies later in life [1, 2]. Among the neurodevelopmental toxicants that received attention in this regard, methylmercury (MeHg) and polychlorinated biphenyls (PCBs) represent a great public health concern, because low-level chronic exposure can occur at various world sites through the consumption of contaminated fish, seafood, and marine mammals meat.

The neurotoxic hazard posed by MeHg and PCBs as well as the unique susceptibility of the developing brain are well documented [3, 4] but no definitive conclusion has yet been reached about the dose-response relationship [5–7], that remain unclear, particularly in relation to concomitant exposures.

Noteworthy, several epidemiologic studies on neurobehavioural endpoints in fish-eating populations at various world sites, including the Faroe Islands [8], the Madeira Island [9], the Brazilian Amazon basin, and more recent investigations on north-american Cree Indian infants, New-Yorkers and Bostonian inhabitants, and European fish consumers, demonstrated mercury-related neuropsychological or neurophysiological adverse effects in the offspring, in the domains of language, attention and memory, as well as visuospatial and motor functions [6, 10, 11].

These effects may have been augmented by concomitant exposure to PCBs, which may affect similar neurobehavioural endpoints [12, 13].

Children exposed to PCBs and related chemicals *in utero* or through breastfeeding have an increased incidence of headaches, cognitive deficits, and significantly delayed psychomotor development [14]. In experimental animal models, a number of long-lasting impairments, for example, learning, behavioural, and neurochemical alterations have been described following maternal exposure to PCBs [15–19].

Because human populations are often exposed to mixtures, as also for MeHg and PCBs in food, raising questions about possible additive, synergistic, or antagonistic effects of the components [20], the potential of these pollutants to interact, and the valuation of their joint effects should be thoroughly investigated, even though it is extremely difficult in epidemiological studies.

To gain this goal, studies investigating biochemical parameters in easily accessible non-neural tissues, which are similar to those targeted by chemicals in the brain (see [21]), may represent a successful approach to developing markers of neurotoxicity, which could be useful in exposed people [22, 23]. In this respect, candidate non-invasive surrogate markers include neurotransmitter receptors, enzymes, and cell signalling components, which are measurable in blood, plasma, lymphocytes, and/or platelets and can be affected by neurotoxicants in these peripheral tissues with changes mirroring those occurring in the brain [21, 24–27].

Examples of these peripheral markers include the cholinergic muscarinic receptors (MRs) in lymphocytes and the enzyme activity of monoamine oxidase-B (MAO-B) in platelets [27–29].

The cholinergic system, essential for normal brain development [30], is a sensitive target for MeHg neurotoxicity [31, 32]. Both *in vitro* and *in vivo* evidences indicate that the cholinergic muscarinic system can be affected by MeHg [25, 33–40], as well as by PCBs during development [41–43]. Notably, coexposure to MeHg and either PCB153 or PCB126 had the same effect on the cerebral MRs as exposure to each compound alone [28]. It has to be considered that changes in levels and activity of MRs have been implicated in the pathophysiology of many major diseases of the CNS [44–48].

Noticeably, in mammalian species, most of the cholinergic components found in the CNS, including MRs, are also expressed in non-neuronal tissues including lymphocytes isolated from peripheral blood, thymus, lymph nodes, and spleen [49]. Accordingly, some neurotransmission parameters measured in rats, including lymphocytes MRs, after the exposure to neurotoxicants and muscarinic drugs [35, 43, 50–52], have been shown to mirror equivalent changes of these biochemical end-points in the CNS, thus providing accessible measures of the same neurochemical endpoints express in the CNS.

Both MeHg and PCBs may also affect the central monoaminergic system as well. In fact, altered dopaminergic neurotransmission has been observed following perinatal exposure to MeHg (0.5 mg/kg/day, GD7-PND7) in rats [53, 54]; moreover, MeHg can stimulate the spontaneous release of monoamines from different experimental CNS tissue preparations [55, 56]. The most consistent neurochemical effects of noncoplanar PCBs have been found to be a reduction in dopamine (DA) concentrations besides an increase in DA concentration both in cells and striatal tissue cultures [57–59] as well as in laboratory animals brains after developmental or adult exposure (for a review, [18]), with an exacerbation of the effects of the two contaminants acting synergistically when coadministrated [58]. On the other hand, in a recent *in vivo* study, we demonstrated that perinatal exposure to MeHg (0.5 mg/kg/day) and/or PCB153 (5 mg/kg/day) given orally to rat dams, affected D1 and D2 receptors in a gender-, time-, and brain area-dependent fashion, without additive effects of the two chemical compounds when administrated in mixture [60].

Noticeably, both MeHg and PCBs may alter the activity of the enzyme monoamine oxidase (MAO), which play an important role in the degradation of monoamine neurotransmitters as well as in the neurochemical regulation of behaviour. Experimental evidences in laboratory animals showed that (i) MeHg inhibited MAO activity both *in vivo *and *in vitro *[61, 62] and (ii) prenatal exposure to PCB77 depressed postnatal development of MAO activity in whole rat brain [63]. Recently, perinatal exposure to MeHg or PCB153 in rats was shown to induce regional alterations of the central dopaminergic and serotonergic systems at weaning, but the combined treatment with both these toxicants does not exacerbate the neurochemical effects of the compounds alone [28].

In humans, the MAO-B isoenzyme is the predominant form in the brain and the sole type present in platelets. The amino acid sequences of MAO-B in both platelets and brain are identical [64], and the biochemical and pharmacological characteristics of the enzyme are also similar in the two tissues [65]. In this respect, platelet MAO has been widely used as a model of central neuronal function and a surrogate marker to investigate neurological and psychiatric disorders [66–70].

With the ultimate goal to identify potential biomarkers of CNS effects, which can be applied as accessible tools to use in environmental medicine for assessing and monitoring specific exposure scenarios, a series of *in vivo* experimental studies have been planned in our laboratory. Specifically, in the attempt to advance knowledge on these biomarkers, a first-step study has been performed in order to evaluate (i) brain and lymphocytes MRs and (ii) cerebral MAO-B activity, investigated in both dams and offspring at weaning, after perinatal exposure to MeHg (0.5 mg/kg/day or 1 mg/kg/day, from gestational day (GD)7 to postnatal day (PD)7, and PCBs (20 mg/kg/day from GD10 to GD16) alone and in combination. Then, in a second step, MRs in lymphocytes (l-MRs) and MAO-B in platelets (p-MAO-B) have been applied in a selected human population, with the aim at supporting (i) the predictive value of these biomarkers and (ii) the relevance of a translational approach in environmental medicine.

## 2. Experimental Protocols

### 2.1. Animal Studies

All experimental procedures involving animals were performed in compliance with the European Council Directive 86/609/EEC on the care and use of laboratory animals. Adult Sprague-Dawley rats (12 females and 4 males, 12 weeks old for each set of experiment) were purchased from Charles River Italia (Calco, Italy) at least 2 weeks before mating and allowed to acclimatize for 3 weeks. Throughout the experiment, animals were kept in an artificial 12 h light : 12 h dark cycle with humidity at 50 ± 10%. Animals were provided rat chow (VRF1 diet) and tap water ad libitum.

To mimic human developmental dietary exposure to this contaminant, rats were exposed to low-to-moderate doses of MeHg and PCB153 *in utero*, through maternal oral consumption.

The experimental regimen comprises 0.5 and 1 mg MeHg/kg (body weight) bw/day, administered to rat dams in the drinking water from gestational day (GD)7 to postnatal (PD)7, and/or PCB153 (20 mg/kg/day) dissolved in corn oil, administered to rats from GD10 to GD16.

The endpoints investigated in rats included total cholinergic MRs and MAO-B activity in cerebellum and total MRs in lymphocytes at weaning (i.e., PD21) both in dams and their offspring.

At the day of sacrifice, rat brains were rapidly dissected on ice to isolate cerebellum from dams and offspring and stored at −80°C until the analyses were performed. Spleens were also collected.

### 2.2. Human Population

The Faroese birth cohort (*n* = 182) was established in 1994–1995 and consisted of singleton term births. The studies adhered to the Declaration of Helsinki and have been performed after approval of the Faroese ethical review committee. All subjects participating in the clinical studies have been included after parental written informed consent. Of the original 182 cohort members, 177 were eligible for participation in the 7-year examinations, and 166 agreed to participate (94%). A total of 159 children (76 boys, 83 girls) completed their examinations at 7 years of age with a voluntary blood sample for the exposure analyses and for the determination of biochemical markers: MAO-B activity in platelet and MR binding in lymphocytes.

### 2.3. MR and MAO-B Determinations in Rat Cerebellum

(i) Total MRs in cerebellum were determined by saturation binding assays using the specific muscarinic antagonist [^3^H]QNB, capable of labeling all subtypes of the MR family uniformly [35, 43, 52]. By these techniques, the receptor density (Bmax, expressed as femtomoles/mg protein) and affinity (defined as the reciprocal of the dissociation constant, Kd) were estimated by nonlinear regression analysis of saturation binding data.

 (ii) MAO-B activity was determined radiochemically as described by Young et al., 1986 [71] using 10 *μ*M ^14^C-PEA as the substrate. Specific MAO-B activity was determined in the presence of 100 *μ*M pargyline hydrochloride. The reaction was started by addition of ^14^C-PEA to 50 *μ*L of tissue homogenate (0.2 mL final volume) and stopped by addition of 0.1 mL citric acid 2 M after a 15-min incubation at 37°C. Deaminated reaction products were extracted into 3 mL toluene-ethylacetate (1 : 1, v/v) and the radioactivity contained in a 1-mL aliquot of the organic phase was counted in scintillation counter. The enzyme activity was expressed as nmol/mg protein/h.

### 2.4. MRs Determination in Lymphocytes

 (i) Rat lymphocytes were obtained from the spleen of controls and differently treated animals [35]. MR density and affinity in rat lymphocytes were determined as described in [52].

 (ii) Human blood was collected in EDTA tubes and immediately processed to isolate lymphocytes for MR binding as previously described [72]. The lymphocytes were resuspended in the freezing solution [90% plasma obtained from autologous blood kept on ice + 10% dimethylsulfoxide (DMSO)]. Immediately after, the cells were gradually frozen at −80°C for 24 h, and thereafter stored in liquid nitrogen.

Muscarinic receptors in humans were determined by binding assays using a single concentration (Kd) of the specific tritiated ligand antagonist [^3^H]QNB for muscarinic receptors in lymphocytes [72]. The specific binding was measured in the presence or absence of atropine. Each sample was assayed in triplicate and data were expressed as fmol/10^6^ cells.

### 2.5. MAO-B Determination in Human Platelet

Human blood was collected in EDTA tubes and immediately processed to isolate platelets for MAO-B activity as previously described [72]. The platelet-rich plasma (PRP) was diluted with 10% DMSO, gradually frozen at −80°C for 24 h, and thereafter stored in liquid nitrogen.

The activity of p-MAO-B was determined radiochemically in duplicate samples as described by Coccini et al., 2002 [66] using [^14^C-PEA] as the substrate. Specific activity was determined in the presence of pargyline hydrochloride. The enzyme activity was expressed as nmol/mg protein/h.

### 2.6. Analytical Measurements of Total Hg Levels in Rats

Measurements of total Hg in cerebellum and blood also complemented the molecular studies to correlate neurochemical changes with the internal doses (see methods in [43, 52, 73]).

### 2.7. Statistical Analysis

Data are presented as the mean ± standard deviation. Data analysis was performed by one-way analysis of variance (ANOVA) followed by Fisher's posthoc test using SPSS statistical software, considering probabilities <0.05 as significant. Statistical comparison of Hg concentrations between groups (Hg versus Hg + PCB153) in brain and blood of dams and both offspring gender was performed using Student's *t* Test.

## 3. Results

The present study assessed (i) firstly, the individual and joint effect of MeHg (0.5 and 1 mg/kg/day, GD7-PD7) and PCB153 (20 mg/kg/day, GD10–GD16), given orally to rat dams, on the activity of cerebellar MAO-B and MRs as well as on lymphocytes MRs, both in dams and offspring 21 days postpartum; and then (ii) MAO-B activity and MRs in 7-year-old children from Faroese birth cohort, wherein prenatal exposure to MeHg and PCB had already been demonstrated [29, 74].

### 3.1. Cerebellar MAO-B Activity

**Figure 1:**
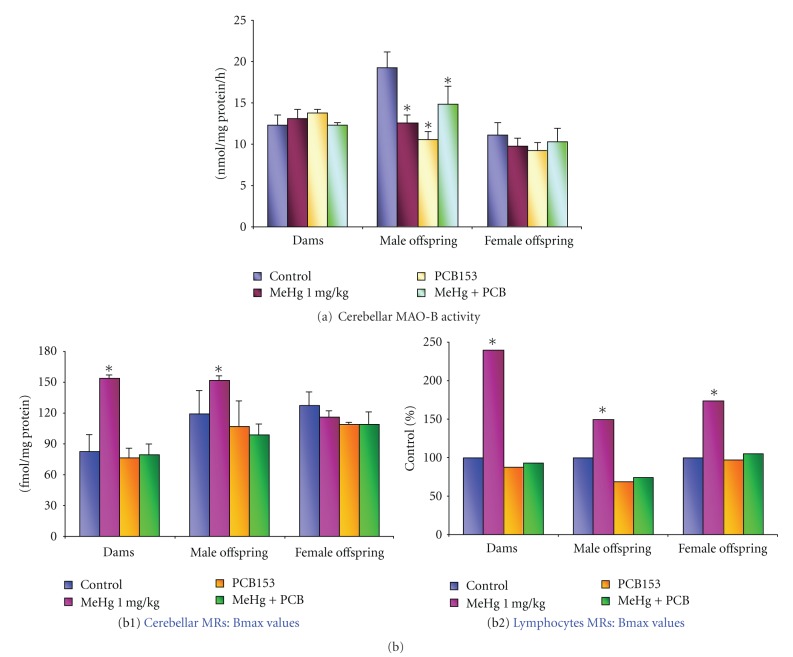
Histograms showing cerebellar MAO-B activity (a) and MRs binding in cerebellum (b1) and lymphocytes (b2) of both dams and 21-day-old male and female rats, perinatally exposed to MeHg (1 mg/kg/day, GD7-PND7) and PCB153 (20 mg/kg/day, GD10–GD16), alone or combined. Data are the mean ± SEM of *n* = 3–9 rats/treatment group/gender. *Significantly different from control group, *P* < 0.05.

Figure 1(a) shows the effects of 1 mg/kg/day MeHg (GD7-PND7) and/or 20 mg/kg/day PCB153 (GD10–GD16) maternal oral treatment on the cerebellar MAO-B activity at PD21 rats, evaluated both in dams and offspring (separately according to gender).

The doses, timing of MeHg and PCB153 administrations, and the time-point (PD21) to determine the neurochemical endpoints were selected from previous experimental data demonstrating changes in rat cerebral MRs density, following the same regimen of perinatal exposure [42]. Furthermore, this treatment protocol neither produced any noxious effect on pregnancy, litter size at birth and ratio between male and female pups, maternal or neonatal body weight increase, nor resulted in any gross abnormality to the pups (data not shown).

In male rat cerebellum, in which the control levels of MAO-B activity (nmol/mg protein/h) were 19.26 ± 2.05, all diverse exposures caused significant changes in the enzyme activity (Figure 1(a)). Particularly, MeHg decreased MAO-B activity by 35%, PCB153 by 45%, and the combined treatment by 25%. On the contrary, in female pups, this neurochemical endpoint was not affected by any treatment (Figure 1(a)), neither was in dams.

Noticeably, no effects on MAO-B activity were observed using the lower MeHg dose (0.5 mg/kg/day from GD7 to PND7) in both male and female offspring (data not presented).

The radiochemical method, employed in the present study to determine MAO-B activity, did not allow the detection of this enzyme in rat platelets.

### 3.2. Cerebellar MRs Density (Bmax)

In accordance to previous investigations [75], all determinations were done at the end of the lactational period (PND21), a time at which the total MR binding sites reach adult levels in rats.

Exposure to the higher dose of MeHg (1 mg/kg/day) significantly enhanced cerebellar MRs density in dams (87%), and, in the offspring, Bmax increase was observed in the male pups only (27%) (Figure 1(b1)). Differently from the response to MeHg, PCB153 did not modify cerebellar MRs density both in adult and offspring. After the concomitant exposure to MeHg (1 mg/kg/day) and PCB153, the MRs density was similar to that detected after the administration of PCB153 alone(Figure 1(b1)).

Noticeably, the lower dose of MeHg (0.5 mg/kg/day) either alone or in combination with PCB153 did not cause any change in MR density both in the mother and offspring cerebella (data not shown).

Cerebellar control Kd values were 0.085 ± 0.02, 0.078 ± 0.01, 0.080 ± 0.01 nM in dams, male, and female offspring, respectively. MeHg and PCB153, alone and in combination, did not affect the dissociation constant values (Kd) measured both in dams and offspring, thus suggesting that the affinity of the ligand [^3^H]QNB for its receptor was not modified by these compounds.

### 3.3. Lymphocytes MRs: Animal Data

Exposure to 1 mg MeHg/kg/day during pregnancy and lactation (from GD7 to PND7) significantly enhanced lymphocyte MR density in both dams and 21 day-old rats, with a more pronounced effect in the mothers (Bmax increase of 139%) than in the male offspring (+49%) and female offspring (+73%) as compared with their respective controls (33 ± 4, 41 ± 8, and  37 ± 4 fmol/million cells, Figure 1(b2)), in accordance with the higher Hg levels detected in the adult blood (11.3 ± 2.26 mg/mL) than in pups (1.35 ± 0.4 mg/L in both genders, Table 1), and in absence of any change in their Kd values (control Kd values were 30 ± 10 nM).

Again, when the lower dose of MeHg (0.5 mg/kg/day) was administered to dams, the density of MRs of all groups did not significantly differ from that of their control groups on day 21 postpartum. This dose of MeHg was also devoid of any effect on the Kd values (data not shown). 

### 3.4. Brain and Blood Hg Levels in Rats

Hg levels were measured in whole brains and blood of both dams and 21-day-old pups (separate accordingly to gender) treated with 1 mg MeHg/kg/day. The Hg concentrations (Table 1) in the brain were about five-fold higher in the mother than in the offspring and resulted to be not affected by the coexposure to PCB153.

Regarding the Hg retention in blood, the levels in the dams were about 10-fold higher than those found in both male and female offspring (Table 1).

### 3.5. Neurochemical Markers in Humans: Lymphocytes MRs and p-MAO-B

**Figure 2:**
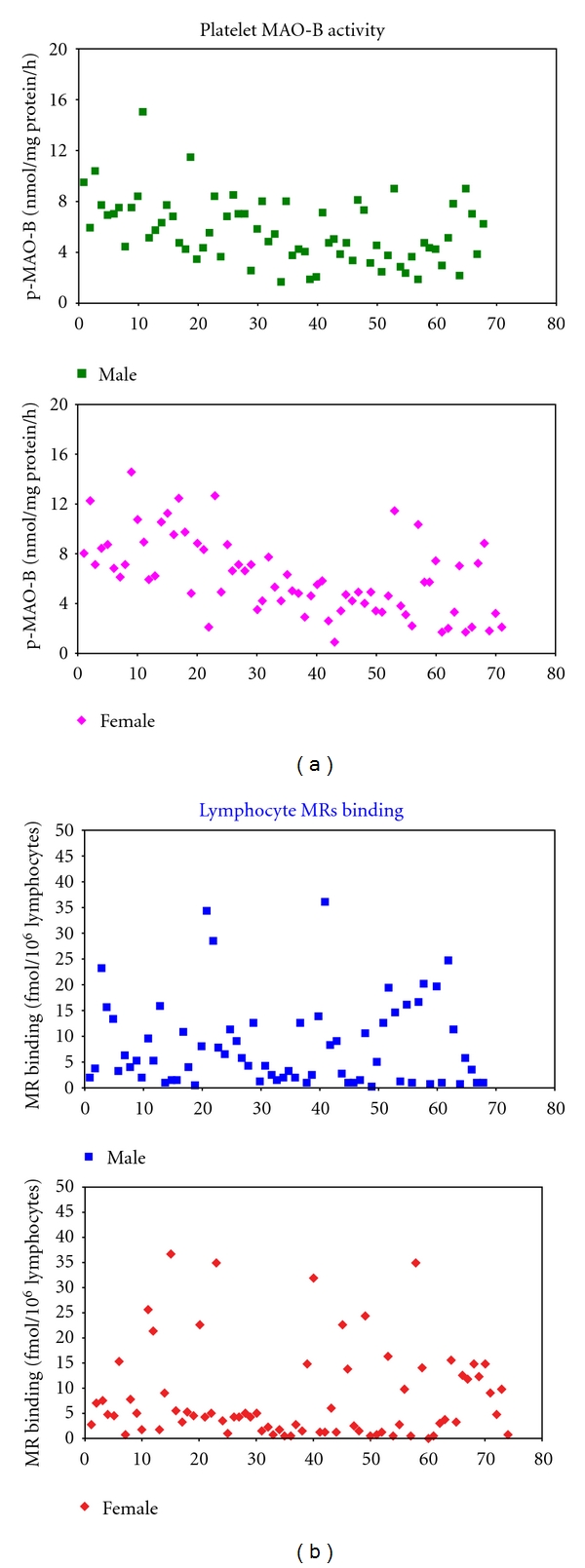
Levels of (a) p-MAO-B activity (*n* = 137) and (b) l-MR binding measured in the blood of both female and male Faroese children. (a) illustrates the individual values of the MAO-B activity in 70 boys (mean ± SD = 6.08 ± 3.12 nmol/mg protein/h, range: 0.95–14.57) and 67 girls (mean ± SD = 5.56 ± 2.55 nmol/mg protein/h, range: 1.57–14.95), respectively. (b) shows the single values of the MR binding in 73 boys (mean ± SD = 8.05 ± 9.13 fmol/10^6^ cells, range: 0.1–36.78) and 66 girls (mean ± SD = 8.09 ± 8.31 fmol/10^6^ cells, range: 0.1–35.91), respectively.

The levels of the lymphocytes MR binding (*n* = 139) and p-MAO-B activity (*n* = 137) measured in the blood of both female and male Faroese children are shown in the Figures 2(a) and 2(b).  Figure 2(b) shows the single values of the MR binding in boys (*n* = 73) and girls (*n* = 66), respectively. The MR binding was similar in boys and girls and ranged from 0.1 to 36.78 fmol/million cells in boys and from 0.1 to 35.91 fmol/million cells in girls.  Figure 2(a) illustrates the individual values of the MAO-B activity in both male (*n* = 70) and female (*n* = 67) children, respectively. Even for this neurochemical endpoint, the data were similar in both genders (ranges: 0.95–14.57 and 1.57–14.95 nmol/mg protein/hr for boys and girls, resp.).

 No correlation (by regression analysis) was found between the two neurochemical biomarkers and neurotoxicant concentrations in blood (pre- and postnatally) [29].

## 4. Discussion

The present study demonstrates in laboratory animals that developmental exposure to MeHg (1 mg/kg/day, GD7-PND7) and PCB153 (20 mg/kg/day, GD10–GD16), alone and in mixture, affects selected endpoints of cholinergic and monoaminergic transmission, namely, cerebellar MAO-B activity and cerebellar and lymphocyte MRs, both in dams and weaning rats, 3 weeks after cessation of maternal dosing.

In the CNS, MAO-B activity was significantly decreased (25–45%) by all treatment types in the cerebellum of male pups only. The combined exposure to MeHg and PCB153 did not exacerbate the neurochemical effects of the individual compounds.

MRs density was significantly affected by MeHg alone both in dams and male offspring cerebella (Bmax enhancement of 87% and 27%, resp.), while, in the coexposed group, no MRs changes were observed.

MRs measured in peripheral lymphocytes also displayed alterations similar to those occurring in the cerebellum, in that MeHg alone significantly enhanced MRs density both in dams and offspring, (Bmax increase of 139%, 49%, and 73% for dams, males, and females, resp.), and, when in combination with PCB153, the latter compound masked the MeHg effects.

In another set of experiments, testing the lower MeHg dose (0.5 mg/kg/day, GD7-PND21), no effects were detected for all the chosen cholinergic and monoaminergic endpoints.

Altogether the present results, obtained both in neural tissue and in peripheral cells, are in agreement with a large body of previous experimental evidence strongly supporting the notion that MR binding is modulated in a similar manner in lymphocytes and cerebral tissues. Supporting examples of this parallel modulation come from cholinergic agonist and antagonist drugs [50] as well as by environmental chemicals, such as MeHg [35] and organophosphorous insecticides [51]. Moreover, the MRs were shown to be similarly modulated in rat brain and in lymphocytes following repeated perinatal MeHg exposure [52].

Again, the present investigation demonstrates that the effects measured in lymphocytes MRs of animals coexposed to MeHg and PCB153 mirror the changes observed in cerebellum, in which the chemical mixture produced no detectable MR alterations.

Based on (i) the results obtained from this laboratory animal study (above reported), (ii) the need to identify early biomarkers of effects for delay neurological outcomes due to environmental neurotoxicants exposure, and (iii) the opportunity to measure these biochemical parameters in easily accessible non-neural tissues, we are prompted to evaluate these peripheral surrogate markers in a specific human casistic.

The human study, performed in a cohort of 7-year-old children with widely different degrees of MeHg exposure, with a built-in control group with low-level exposure, indicates that children exhibited homogeneous distributions of the two neurotoxicity biomarkers with no changes associated with increased concentrations of mercury or coexposure with PCB congeners and with no clear association with outcomes of clinical neurobehavioral testing.

These results seem to support the notion that p-MAO-B and l-MRs are not adequately sensitive markers to early detect the subclinical outcomes of MeHg and/or PCBs at low/moderate exposure doses.

Contrarily, recent investigation by Stamler et al. 2006 [68] in a Canadian fish-eating population inhabiting the St. Lawrence River (Lake St. Pierre, Quebec) demonstrated a MeHg-induced reduction in MAO-B activity, associated with blood-Hg concentrations above 3.4 *μ*g/L.

On the other hand, in animal studies, the used perinatal dose of 1 mg MeHg/kg/day markedly affected the rat cholinergic and aminergic systems, while, contrarily, the lower dose of 0.5 mg MeHg/kg/day was totally ineffective. After the former treatment, cerebral Hg levels in weaning rats were 1.5–2.8 ppm and blood Hg levels were 1.3 ± 0.4 *μ*g/mL (in both pup genders). These latter data are clearly much higher (250 to 40 times) compared to humans blood Hg levels, for instance, 1300 ppb (in rat blood at PD21) versus 27.6 and 5.27 ppb in cord blood and 7-year-old children blood of the present cohort, respectively [29]. These observations put forward that MeHg-exposure levels may affect the alterations in neurotransmission pathways and, consequently, the changes in both central and peripheral markers. Furthermore, other confounding factors, such as genetic variables, may also be to taken into consideration for their potential impact on the background levels of these biomarkers [76–79].

Moreover, since the precise time point at which these peripheral biomarkers become predictive of neuropsychological outcomes (noticeable later in life) has not been yet clarified, this should be investigated correlating these parameters (neurochemical and neuropsychological) in an early (e.g., cord blood) and late time point manner within the same subject. In this way, one could try to minimize the influence of the blood cell turnover on the levels of these cell-coupled surrogate markers, in that, because of this mechanism, the response to a moderate environmental toxicant exposure may not reflect the possible effect at vulnerable time windows (e.g., *in utero* period) that may cause permanent changes in neuropsychological outcomes.

Nevertheless, a great body of epidemiological evidence indicated the suitability of these biomarkers when employed as peripheral indicators of abnormal behaviour/personality or changes in response to a diagnosed pathological conditions [80–82], and drug dependence [66, 83–85].

Therefore, even though all these above-reported findings and the available bulk of literature seem to be sometimes and somewhere controversial, integrated approaches combining biochemical markers in combination with neurophysiological and behavioral assays could represent a valuable methodological approach by which human neurotoxicity assessment may become more focused.

## Figures and Tables

**Table 1:** Total Hg levels in blood and brain of rats perinatally treated with 1 mg MeHg/kg bw/day (GD7-PD7) and/or 20 mg PCB153/kg bw/day (GD10–GD16), alone and in combination.

	Blood Hg (*μ*g/ml)			Brain Hg (*μ*g/g)		
	*Dams*	*Offspring*		*Dams *	*Offspring*
		*Male*	*Female*		*Male*	*Female *
Control	0.017 ± 0.004 *n* = 3	0.003 ± 0.001 *n* = 4	0.005 ± 0.004 *n* = 4	0.013	0.002	0.003
MeHg	11.33 ± 2.26 *n* = 4	1.35 ± 0.40 *n* = 4	1.31 ± 0.40 *n* = 4	7.53	1.67 ± 0.43 *n* = 4	1.52 ± 0.33 *n* = 4
MeHg + PCB153	10.74 ± 1.95 *n* = 5	1.33 ± 0.33 *n* = 5	1.26 ± 0.20 *n* = 5	8.78	2.80 ± 1.81 *n* = 6	1.63 ± 0.20 *n* = 6
PCB153	0.012 ± 0.001 *n* = 5	0.003 ± 0.001 *n* = 4	0.002 ± 0.001 *n* = 5	0.026	0.024 ± 0.039 *n* = 4	0.014 ± 0.024 *n* = 6

Data are the mean ± S.D.
